# Cytoarchitectonical analysis and probabilistic mapping of two extrastriate areas of the human posterior fusiform gyrus

**DOI:** 10.1007/s00429-012-0411-8

**Published:** 2012-04-10

**Authors:** Julian Caspers, Karl Zilles, Simon B. Eickhoff, Axel Schleicher, Hartmut Mohlberg, Katrin Amunts

**Affiliations:** 1 Institute of Neuroscience and Medicine (INM-1, INM-2), Research Centre Jülich, 52425 Jülich, Germany; 6 Institute of Clinical Neuroscience and Medical Psychology, Heinrich-Heine-University Düsseldorf, 40225 Düsseldorf, Germany; 2 C. and O. Vogt Institute for Brain Research, Heinrich-Heine-University Düsseldorf, 40225 Düsseldorf, Germany; 3 JARA-BRAIN, Jülich-Aachen Research Alliance, 52425 Jülich, Germany; 4 Department of Psychiatry, Psychotherapy, and Psychosomatics, RWTH Aachen University, 52074 Aachen, Germany

**Keywords:** Cytoarchitecture, Probabilistic mapping, Fusiform gyrus, Lateral occipital complex (LOC), Fusiform face area (FFA), Visual word-form area (VWFA)

## Abstract

The human extrastriate visual cortex comprises numerous functionally defined areas, which are not identified in the widely used cytoarchitectonical map of Brodmann. The ventral part of the extrastriate cortex is particularly devoted to the identification of visual objects, faces and word forms. We analyzed the region immediately antero-lateral to hOc4v in serially sectioned (20 μm) and cell body-stained human brains using a quantitative observer-independent cytoarchitectonical approach to further identify the anatomical organization of the extrastriate cortex. Two novel cytoarchitectonical areas, FG1 and FG2, were identified on the posterior fusiform gyrus. The results of ten postmortem brains were then registered to their MRI volumes (acquired before histological processing), 3D reconstructed, and spatially normalized to the Montreal Neurological Institute reference brain. Finally, probabilistic maps were generated for each cytoarchitectonical area by superimposing the areas of the individual brains in the reference space. Comparison with recent functional imaging studies yielded that both areas are located within the object-related visual cortex. FG1 fills the gap between the retinotopically mapped area VO-1 and a posterior fusiform face patch. FG2 is probably the correlate of this face patch.

## Introduction

The human visual cortex can be divided into the primary visual or striate cortex (V1 or BA17) (Brodmann 1909) and the adjoining extrastriate cortex. The extrastriate cortex covers the largest part of the occipital lobe and extends into the posterior parietal and temporal regions. Here, two processing pathways have been described, the dorsal pathway or “where”-stream for spatial localization and visually guided action, and the ventral pathway or “what”-stream, which is involved in object, color and shape recognition (Mishkin and Ungerleider 1982; Ungerleider and Haxby 1994; Eickhoff et al. 2008). During the last decades, a high degree of functional heterogeneity within the ventral visual cortex has been revealed by functional imaging, giving evidence for several functionally specialized areas, e.g. the fusiform face area (FFA, Kanwisher et al. 1997; Grill-Spector et al. 2004; Kanwisher and Yovel 2006; Weiner and Grill-Spector 2010), the parahippocampal place area (PPA, Epstein et al. 1999; Epstein 2008), the visual word-form area (VWFA, Cohen et al. 2000; Wandell et al. 2012) or the extrastriate body area (EBA, Downing et al. 2001; Peelen and Downing 2005; Weiner and Grill-Spector 2011b). Combined functional and cytoarchitectonical studies of extrastriate areas co-registered in the same reference space are rare (Wohlschläger et al. 2005; Wilms et al. 2010).

**Fig. 1:**
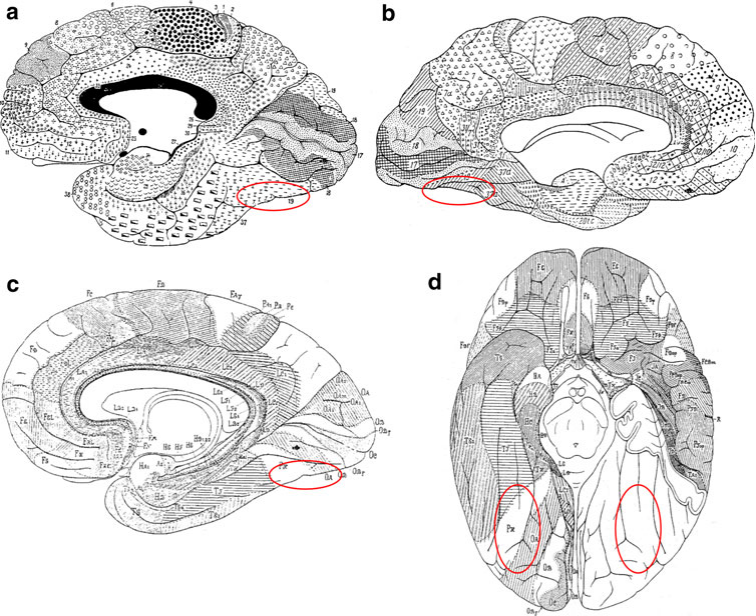
Cytoarchitectonical maps by **a** Brodmann (1909), **b** Sarkisov et al. (1949) and **c**, **d** von Economo and Koskinas (1925); **a**–**c** medial view and **d** ventral view. The regions on the posterior fusiform gyrus, which were investigated in the present study, are marked by *red ellipses*

Conventionally, four criteria have been used to distinguish the visual areas: (1) retinotopy, (2) functional properties, (3) histology and (4) intracortical connections (Clarke and Miklossy 1990; Felleman and van Essen 1991; Sereno et al. 1995; Zilles and Clarke 1997; Tootell et al. 2003; Kolster et al. 2010). The classical anatomical maps from the beginning of the last century (Brodmann 1909; von Economo and Koskinas 1925; Sarkisov et al. 1949) have several disadvantages when attempting to establish functional–structural relationships: Most classical anatomical maps only show a principal tripartition of the visual cortex with V1 (BA17/O_C_), V2 (BA18/O_B_) followed by a single large area (BA19/O_A_) (Brodmann 1909; von Economo and Koskinas 1925) (Fig. 1). Functionally and also histologically, however, it becomes evident that BA19/O_A_ comprises a variety of functional and cytoarchitectonical areas within its ventral and dorsal parts (Zeki 1969; van Essen 1979; Braak 1980; Tootell et al. 1996; Zilles and Clarke 1997; Orban et al. 2004; Malikovic et al. 2007; Rottschy et al. 2007). Moreover, all classical anatomical maps are reported as schematic 2D hand drawings of the cortical surface, which rarely provide information about cortical areas within the sulci and contain no information about stereotaxic location or interindividual variability of cortical areas. Finally, these maps are established on the basis of subjective criteria for the definition of cortical borders.

These shortcomings have prompted the development of probabilistic cytoarchitectonical maps (Amunts and Zilles 2001; Zilles et al. 2002; Zilles and Amunts 2010). The maps are based on the observer independent, statistically testable analysis of postmortem brains (Schleicher et al. 2005, 2009), and contain information about stereotaxic position and intersubject variability of cortical areas in the Montreal Neurological Institute (MNI) reference space (Evans et al. 1992). The maps help to probabilistically identify the cytoarchitectonical correlate of neuroimaging activations seen in functional MRI, positron emission tomography (PET), magneto- and electroencephalography (MEG/EEG) using standard analyses packages (Eickhoff et al. 2005; Zilles and Amunts 2010). As part of this atlasing project, probabilistic maps of the visual cortex have already been defined for the primary visual area hOc1 (BA17) (Amunts et al. 2000), for secondary hOc2 (BA18) (Amunts et al. 2000), and for tertiary hOc3v, hOc4v (Rottschy et al. 2007) and hOc5 (Malikovic et al. 2007).

Since cytoarchitectonical maps of the ventral visual areas anterior to hOc4v are presently not available, the aim of our study was to investigate the structural organization of this region on the posterior fusiform gyrus.

## Materials and methods

### Histological processing

**Fig. 2:**
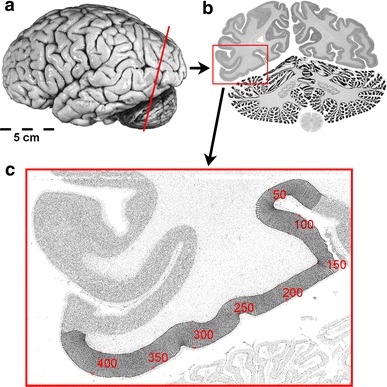
Histological procedure. **a** Postmortem brain sectioned in coronal plane. **b** Cell body-stained coronal section (20 μm) at the position marked in **a**. The region of interest (ROI) is labeled by the *red box*. **c** Inverted gray level index (GLI) image of the ROI with traced outer and inner cortical contours and curvilinear trajectories along the cortical ribbon. *Red numbers* and *trajectories* indicate the position along the cortical ribbon

Ten human postmortem brains (Amunts et al. 2000), five males and five females (Table 1), were obtained from the body donor program of the Institute of Anatomy, University of Düsseldorf. One brain came from a subject with transitory motor disturbance (brain 3), all other donors had no history of neurological or psychiatric diseases. Handedness of the subjects was unknown. We may assume that most of the subjects were right-handed supposing a prevalence of left-handedness of <10 % in the general population (Annett 1973). The brains were removed from the skulls within 8–24 h after death and fixated in 4 % formalin or Bodian’s fixative for at least 6 months. To document brain size and shape before the inevitable distortions by histological processing occurred, each brain was scanned using a T_1_ weighted 3D-FLASH sequence (flip angle 40°, TR = 40 ms, TE = 5 ms) implemented on a Siemens 1.5T scanner (Erlangen, Germany). Subsequently, the complete brains were embedded in paraffin and serially cut into coronal sections of 20 μm thickness. Every 15th section was mounted on glass slides and silver stained for cell bodies (Merker 1983) to achieve a high contrast between darkly stained neuronal perikarya and unstained neuropil. Every fourth stained section, i.e. every 60th section of the series of sections, was examined, resulting in a distance of 1.2 mm between the analyzed sections (Fig. 2).

**Table 1:** List of the ten postmortem brains

Case	Age (years)	Gender	Cause of death	Fresh weight (g)
1	79	F	Carcinoma of the bladder	1,350
2	56	M	Rectal carcinoma	1,270
3	69	M	Vascular disease	1,360
4	75	M	Acute glomerulonephritis	1,349
5	59	F	Cardiorespiratory insufficiency	1,142
6	54	M	Cardiac infarction	1,622
7	37	M	Cardiac arrest	1,437
8	72	F	Renal arrest	1,216
9	79	F	Cardiorespiratory insufficiency	1,110
10	85	F	Mesenteric infarction	1,046

### Detection of cortical borders

Cytoarchitectonical analysis was performed using a quantitative method for observer independent and statistically testable detection of cortical borders (Zilles et al. 2002; Schleicher et al. 2005, 2009). First, rectangular regions of interest covering the posterior fusiform gyrus and neighboring cortex were defined in the histological sections and digitized using a microscope with a scanning stage (KS400; Zeiss, Oberkochen, Germany) and a CCD camera (Sony, Tokyo, Japan; resolution 1.01 × 1.01 μm²/pixel) (Fig. 2b). The digitized sections were then transformed into gray level index (GLI) images, in which pixel values represent the volume fraction of cell bodies in the corresponding square measuring fields of 20 × 20 μm² each (Schleicher et al. 2000, 2005). If the GLI amounts to 20 %, for example, 80 % of the volume in a measuring field are occupied by neuropil (dendrites, axons, processes of glial cells and blood vessels).

Equidistant GLI profiles were extracted along curvilinear trajectories oriented perpendicular to the cortical layers and running from an interactively defined outer contour between layer I and layer II to an inner contour between layer VI and the white matter (Fig. 2c). These profiles represent the course of the regional cell density from superficial (outer contour) to deep (inner contour). To compensate for variations in cortical thickness, we resampled each profile with linear interpolation to a standard length corresponding to a cortical thickness of 100 %. The shape of each GLI profile was quantified by a vector consisting of ten features based on central moments, which were also used for previous cytoarchitectonical studies (e.g. Amunts et al. 2000; Amunts and Zilles 2001; Zilles et al. 2002). These features were: the mean GLI value, the position of the center of gravity on the profile curve (cortical depth), the standard deviation of the mean GLI (indicating the variability of the GLI throughout all layers), skewness and kurtosis of the profile curve and the respective features from the profile’s first derivative (Schleicher et al. 1999).

**Fig. 3:**
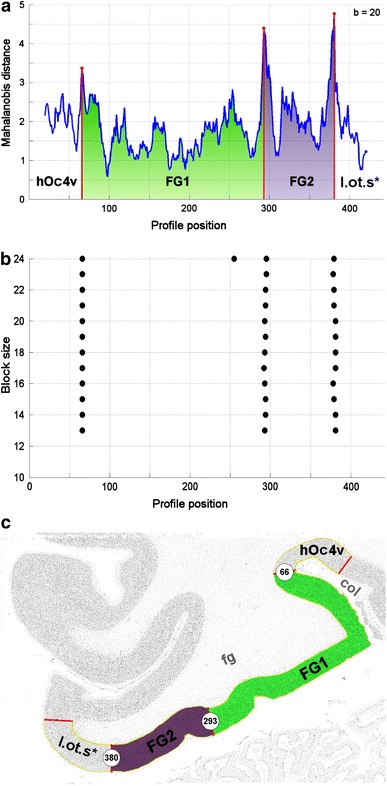
Mapping procedure in the ROI as shown in Fig. 2. **a** Mahalanobis distance function illustrating distances (ordinate) between blocks of GLI profiles for blocksize = 20, and trajectory positions (abscissa). Significant local maxima are *marked in red*. Corresponding cortical areas are labeled. **b** Positions of significant maxima in the distance functions (abscissa) plotted against block sizes *b* (ordinate: 10 ≤ *b* ≤ 24). **c** Inverted GLI image of the ROI from Fig. 2 with marked cortical borders and labeled cortical areas. Only those profile positions were accepted as cortical borders, which showed the maxima in the Mahalanobis distance functions for different block sizes (compare *b*). *col* collateral sulcus, *fg* fusiform gyrus, *FG1* area 1 of the fusiform gyrus, *FG2* area 2 of the fusiform gyrus, *hOc4v* (Rottschy et al. 2007), l.ot.s.* unmapped area in the lateral occipital cortex. The asterisk indicates that this area was not completely mapped

Differences in shape between GLI profiles indicate differences in cytoarchitecture and were quantified as the Mahalanobis distance (Mahalanobis et al. 1949; Bartels 1979) between the respective feature vectors of neighboring blocks of profiles (Schleicher et al. 2005) at every position along the cortical ribbon (Fig. 3). To assure reliability, the procedure is repeated for different block sizes ranging from 8 to 24 profiles per block. Areal borders are expected at positions where the distance function shows local maxima corresponding to a great dissimilarity in laminar pattern between adjacent blocks of profiles. These maxima were detected and their significance evaluated by the Hotelling’s *T*² test with Bonferroni correction for multiple comparisons (Fig. 3b). Cortical borders were confirmed, if they were consistently present at the same position across several block sizes, and if the positions were found at comparable sites in adjacent sections.

### Computation of probabilistic cytoarchitectonical maps

3D reconstructions of the histological volumes were computed using the following three datasets: (i) the previously ascertained 3D-MRI scan, (ii) images of the paraffin block face obtained during sectioning for the precise alignment of the histological sections and (iii) the digitized images of the cell body-stained sections (Amunts et al. 2004).

The defined borders of the cortical areas were interactively traced on the corresponding sections of the 3D-reconstructed brains. The histological volumes were then spatially normalized by registration to the stereotaxic space of the Montreal Neurological Institute (MNI) (Evans et al. 1992) using a combination of affine transformations and nonlinear elastic registration (Hömke 2006). To keep the anterior commissure as the origin of the coordinate system and for consistency with previous cytoarchitectonical studies (e.g. Amunts et al. 2005; Rottschy et al. 2007; Caspers et al. 2008; Scheperjans et al. 2008; Kurth et al. 2010), data were shifted by 4 mm caudally (*y*-axis) and 5 mm dorsally (*z*-axis) to the ‘anatomical MNI-space’ (Amunts et al. 2005). Corresponding areas of the ten brains were superimposed and a probabilistic map was generated for each area (Zilles et al. 2002). These maps indicate for each voxel of the reference brain the relative frequency with which a respective area was found at that position (Eickhoff et al. 2007; Zilles and Amunts 2010).

The probability maps of all areas in the ventral visual cortex were visualized as a continuous, non-overlapping map of this region by generating a maximum probability map (MPM). The MPM was computed by comparing the probabilities of all areas in each single voxel and by assigning to each voxel the cytoarchitectonical area with the highest probability (Eickhoff et al. 2005, 2006). If two or more areas showed equal probabilities in a single voxel, this voxel was assigned to the area with the highest average probability of the directly adjacent voxels.

### Analysis of volumes

The volumes of the cytoarchitectonical areas were calculated for each hemisphere separately based on area measurements in the individual histological sections, section thickness and distance between the measured sections as well as the shrinkage factor of each brain. Shrinkage factors were determined as the ratio between the fresh volumes of the brains and their volumes after histological processing (Amunts et al. 2005). The volumetric data were then analyzed for interindividual and interhemispheric differences using a repeated measurement analysis of variance (ANOVA) with the following design: between-subject factor: gender; within-subject factors: area and side; blocking factor: subject.

## Results

**Fig. 4:**
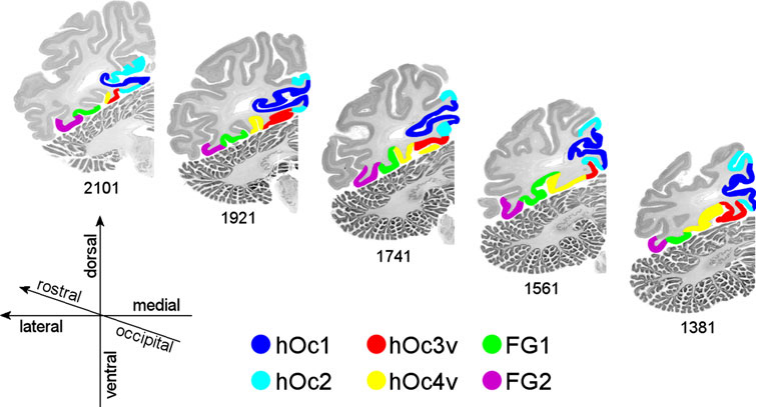
A rostro-caudal sequence of five coronal MRI sections through the left hemisphere of one single brain. Section numbers are indicated below the sections. The cortex of visual areas hOc1, hOc2, hOc3v, hOc4v, FG1 and FG2 is labeled in *different colors*. Distance between sections 3.6 mm

Two cytoarchitectonically distinct areas, FG1 and FG2, were identified antero-lateral to hOc4v (Rottschy et al. 2007) on the posterior fusiform gyrus (Fig. 4). The term “FG” was used for “fusiform gyrus”. This allows a neutral, macroanatomical nomenclature. The different areas of the fusiform gyrus were labeled by numbers instead of more descriptive labels like “medial” or “lateral”. This avoids future inconsistencies, as the total number of cytoarchitectonical fusiform areas and their relative spatial arrangement are not yet known. Since the cytoarchitectonical areas described here were not previously identified, any assignment to areas of Brodmann (1909) or von Economo and Koskinas (1925) was not meaningful. Asterisks behind the names of two other areas (col.s.* and l.ot.s.*) indicate that these areas were not completely mapped in the present observation.

The more medial area FG1 was located immediately lateral to the rostral portion of area hOc4v. It was found on the medial half of the posterior part of the fusiform gyrus and extended on the lateral bank of the collateral sulcus. FG2 was located lateral to FG1 on the lateral half of the fusiform gyrus and on the lateral occipitotemporal sulcus. Rarely, minor parts of FG2 reached onto the inferior temporal gyrus. FG2 extended more rostrally than FG1. The rostral part of FG2 covered nearly the full width of the fusiform gyrus.

### Cytoarchitecture

**Fig. 5:**
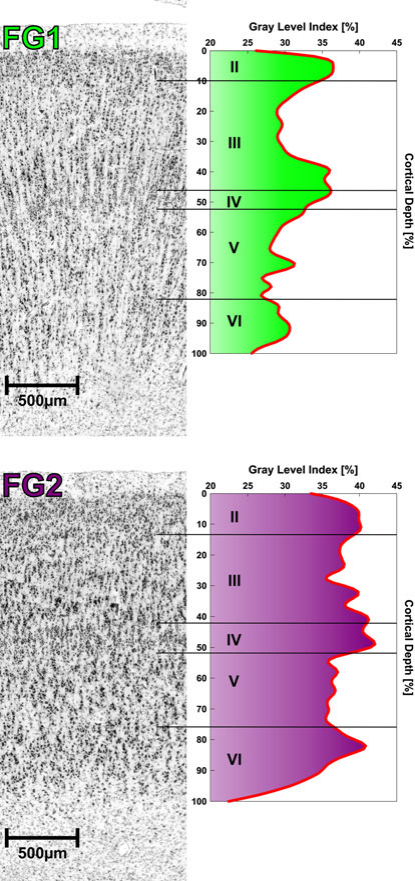
Cytoarchitecture of areas FG1 and FG2 with the corresponding GLI profiles. *Roman numerals* indicate cortical layers

Both areas belong to the homotypical isocortex with an inner granular layer IV but with further distinctive cytoarchitectonical features:

FG1 was characterized mainly by a marked columnar arrangement of small pyramidal cells and a lower cell density in layer IV (Fig. 5) when compared to neighboring areas. Layer II showed a rather low density of cells and smooth transition into a layer III of low to moderate cell density. The sizes of pyramidal cells in layer III were small with no superficial to deep increase. These pyramidal cells were arranged in clearly visible cell columns, a feature that was unique to FG1 compared to the adjacent areas. Layer IV of FG1 was thin, but clearly delineable from adjacent layers IIIc and V. Pyramidal cells in layer V were small and showed a columnar arrangement as well. The cell sparse layer VI had no conspicuous border with lower layer V. The border between cortex and white matter was blurred.

FG2 showed large pyramidal cells in lower layer III and a prominent layer IV as characteristic features (Fig. 5). A columnar arrangement of pyramidal cells was not found, neither in layer III nor in layer V. Layer II of FG2 had a rather high cell density with a clearly delineable border to the cell sparse layer IIIa. Large, typical pyramidal cells of layer III were primarily found in sublayers IIIb and IIIc with a slight increase of cell sizes in deep sublayer IIIc. This sublayer often merged with the broad and cell dense layer IV. Layer V consisted of equally distributed pyramidal cells, which were smaller than those in lower layer III but larger than those of FG1. Layer V was clearly delineable from the cell dense layer VI, which again had a distinct border to the white matter.

**Fig. 6:**
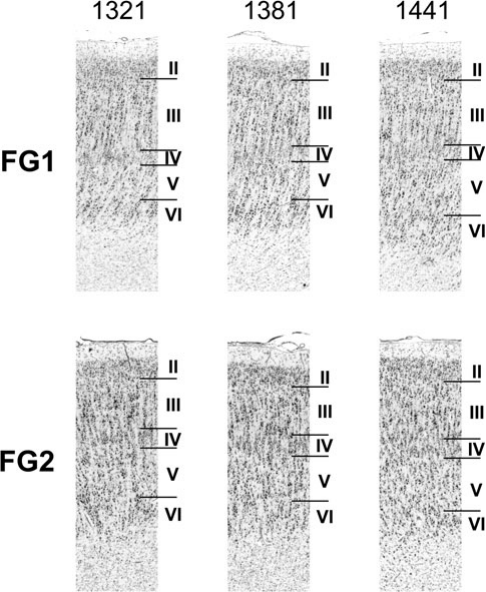
Cytoarchitectonical variability of areas FG1 and FG2 between slices. For each area, segments of three consecutive sections from one single hemisphere (*brain 8 left*) are shown. *Numbers above images* indicate the section number. *Roman numerals* indicate cortical layers

**Fig. 7:**
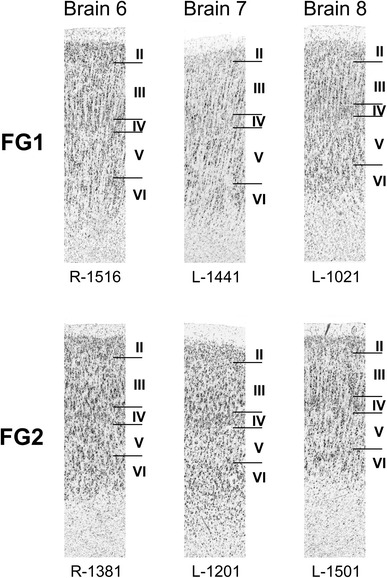
Cytoarchitectonical variability of areas FG1 and FG2 between subjects. For each area, segments of sections from three different brains are shown. Brain number is indicated above the images. An ‘R’ or ‘L’ below indicates the hemisphere (*right or left*) and the following number labels the section number. *Roman numerals* indicate cortical layers

To demonstrate the variability of these cytoarchitectonical features of FG1 and FG2 along the rostro-caudal extent, Fig. 6 illustrates three representative sections of a single brain. The intersubject variability of cytoarchitectonical features is illustrated by sections of three different brains (Fig. 7). Figures 6 and 7 highlight that the main characteristic features of each area, e.g. visible or not visible columnar arrangement of pyramidal cells, prominent or inconspicuous subdivisions of layer III, and width and cell density in layer IV, could be found as distincitive features at all sectioning levels and in all brains studied.

**Fig. 8:**
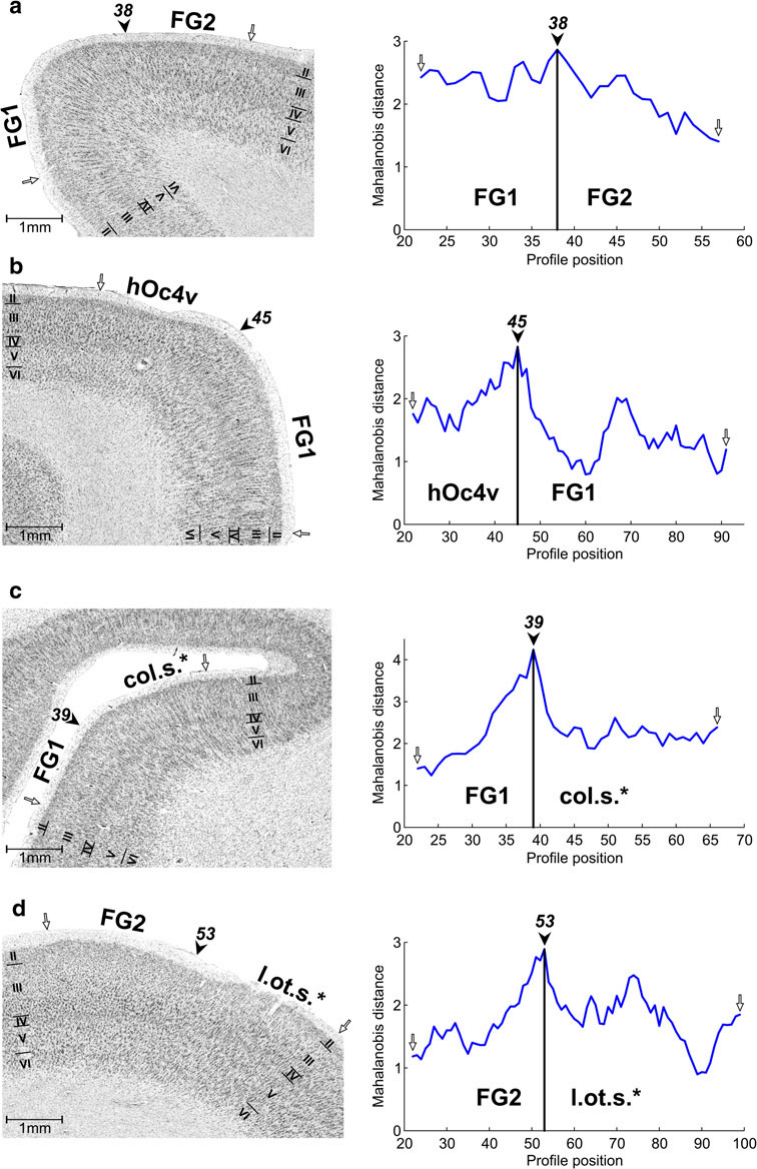
Cytoarchitecture and corresponding Mahalanobis distance functions (block size = 22) of adjoining cortical areas. Cortical borders, which correspond to the maxima in the distance functions, are marked with a *black arrowhead* and the respective profile position on the histological image. *White arrows* indicate the beginning and endpoints of the distance functions. *Roman numerals* label cortical layers. **a** Border between FG1 and FG2. **b** Border between FG1 and hOc4v. **c** Border between FG1 and the medially adjoining area in the collateral sulcus (col.s.*), anterior to hOc4v. **d** Border between FG2 and the laterally adjoining area in the lateral occipitotemporal sulcus (l.ot.s.*). The *asterisks* indicate that these areas were not completely mapped

FG1 and FG2 differed not only by their cytoarchitecture (Fig. 8a) but could also be separated from adjoining cortical areas (see Table 2 for an overview):

**Table 2:** Cytoarchitectonical features of FG1, FG2 and the adjoining areas

		hOc4v	col.s.*	FG1	FG2	l.ot.s.*
II	Cell density	●●●	●●	●●	●●●	●●
III	Pyramidal size	●●	●●	●	●●	●●●
	Pyramidal arrangement	Superf. to deep increase	Deep IIIc	Cell columns	IIIb and IIIc	IIIc
IV	Width	●●	●●	●	●●●	●●
	Cell density	●●●	●●	●	●●●	●
V	Cell density	●●	●●	●	●●	●
VI	Cell density	●●●	●●	●	●●●	●

Feature intensity is coded with black dots: ●●● high, ●● medium and ● low. The area on the collateral sulcus medially adjoining FG1 is labeled with col.s.*, the area laterally adjoining FG2 is labeled with l.ot.s*. An asterisk indicates that these areas are not mapped in detail

Posteriorly, FG1 immediately adjoined hOc4v, which was characterized by a more cell dense layer II with a well-defined border to layer III (Fig. 8b). This latter layer showed larger pyramidal cells with a characteristic superficial to deep increase of cell sizes and no clear arrangement in cell columns. Layer IV provided the most pronounced distinction as it was prominent and had a higher cell density in hOc4v when compared to FG1. Cells in layer V were also larger in hOc4v but not densely packed. Layer VI of hOc4v again showed a higher cell density and a better defined border to the white matter.

In the more rostral parts, FG1 was followed medially by a new, not further analyzed area col.s.*, which was found anterior to hOc4v (Fig. 8c). col.s.* showed medium-sized and densely packed pyramidal cells in layer III, particularly in sublayer IIIc, with no columnar arrangement. Layer IV of col.s.* was more cell dense and slightly thicker than that of FG1, layer V showed a higher cell density, and layer VI had a distinct border to the white matter.

The likewise not yet completely mapped area l.ot.s.* was found lateral to FG2. Area l.ot.s.* extended into the lateral occipitotemporal sulcus and on the inferior occiptal and inferior temporal gyrus. l.ot.s.* showed cell sparse sublayers IIIa and IIIb with large pyramidal cells only in lower IIIc (Fig. 8d). Layer IV of this area, was less prominent than in FG2 and difficult to delineate from layer III. The cell density of layer V in l.ot.s.* was lower than in FG2, and the border between layer V and layer VI was blurred.

### Volumes and stereotaxic location

The statistical analysis of the histological volumes of FG1 and FG2 (Table 3) revealed a significant size difference between both areas (*F* = 25.547, *p* = 0.01) in favor of FG2. There were no significant effects of the factors side (*F* = 2.990, *p* = 0.122), gender (*F* = 1.784, *p* = 0.218) and no significant effects of the interactions between area and side (*F* = 0.011, *p* = 0.920), between area and gender (*F* = 0.254, *p* = 0.628), between side and gender (*F* = 0.380, *p* = 0.555) and between area, side and gender (*F* = 0.001, *p* = 0.981).

**Table 3:** Histological volumes (mm³) of the areas in a sample of ten brains, corrected for shrinkage

	Mean	SD
FG1		
Left hemisphere	1,091	333
Right hemisphere	886	322
FG2		
Left hemisphere	1,617	554
Right hemisphere	1,430	575

Volumes did not differ between the hemispheres (*p* > 0.05)

The coordinates for the centers of gravity of areas FG1 and FG2 in anatomical MNI space are given in Table 4. A slight difference of the anterior–posterior location was noted as both areas were located more rostrally in the right hemisphere. This interhemispheric shift has already been reported for other striate and extrastriate areas in previous studies (Amunts et al. 2000; Rottschy et al. 2007), and reflects the marked asymmetry in position of the occipital poles in the MNI single-subject template.

**Table 4:** Coordinates of the centers of gravity in anatomical MNI space for the probability maps  (PMap) and the maximum probability map (MPM) of areas FG1 and FG2

	*x*	*y*	*z*
FG1			
Left hemisphere			
PMap	−32.3	−76.3	−8.8
MPM	−29.7	−75.6	−8.6
Right hemisphere			
PMap	33.6	−73.6	−10.1
MPM	33.4	−73.1	−10.8
FG2			
Left hemisphere			
PMap	−42.7	−72.3	−12.6
MPM	−41.2	−74.3	−12.3
Right hemisphere			
PMap	42.0	−70.7	−13.3
MPM	42.1	−72.1	−13.7

### Probability map and maximum probability map

**Fig. 9:**
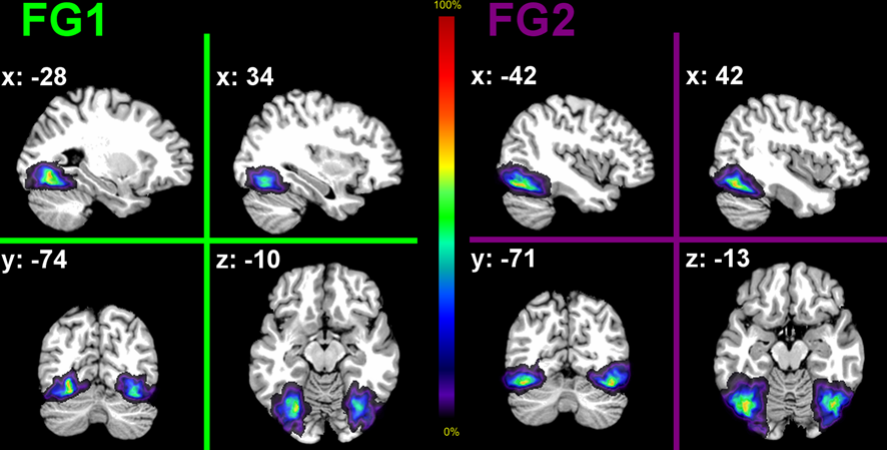
Probability maps of areas FG1 and FG2 in the MNI single-subject reference template in selected sagittal (*top*), coronal (*bottom left*) and horizontal (*bottom right*) sections. The degree of overlap is *color coded* (see *color bar*). Stereotaxic coordinates of the illustrated sections in anatomical MNI space are denoted in the *top left corners* of each map

Probability maps (PMaps) were generated for each FG area by superimposing the spatially normalized representations of the individual subjects (Fig. 9). The PMaps reflect the probability of observing the respective area in single voxels of the MNI space in our sample of postmortem brains. The probability maps for FG1 and FG2 barely showed regions with high probabilities for one area (overlap of 9 or 10 brains), but broad regions with low probabilities (overlap of 1 or 2 brains) in the periphery of each of the FG areas. This reflects a high intersubject variability of both areas, which is much higher compared to early visual areas hOc1 and hOc2 (Amunts et al. 2000).

**Fig. 10:**
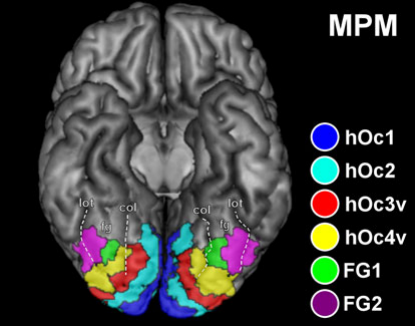
Maximum probability map (MPM) of the visual cortex including hOc1 (*blue*), hOc2 (*cyan*), hOc3v (*red*), hOc4v (*yellow*), FG1 (*green*) and FG2 (*violet*) projected on a 3D rendering of the MNI single-subject reference template without the cerebellum. Basal view is shown. *Dashed lines* highlight the position and extent of sulci delimiting the fusiform gyrus. *fg* fusiform gyrus, *col* collateral sulcus, *lot* lateral occipitotemporal sulcus

Consequently, there was substantial overlap between the probabilistic maps of FG1 and FG2 and also adjoining area hOc4v. To assign the most likely area to each single voxel, an MPM of the visual cortex (comprising areas hOc1, hOc2, hOc3v, hOc4v, FG1 and FG2) was computed (Fig. 10). The MPM represents a contiguous, non-overlapping parcellation of this region. It hence bears a close conceptual resemblance to presentations in classical brain maps. Importantly, however, the MPM does not show the parceling of an exemplary or “typical” hemisphere as do previous architectonic brain maps (Brodmann 1909; von Economo and Koskinas 1925), but reflects the most likely area based on a sample of ten brains and represented in each voxel of the reference space.

## Discussion

This study reports the microscopical features and stereotaxic locations of two previously unknown cytoarchitectonical areas, FG1 and FG2, of the ventral human visual cortex. Our mapping study used quantitative, statistically testable cytoarchitectonical criteria (Schleicher et al. 2005), to overcome uncertainties caused by pure visual inspection (Brodmann 1909; von Economo and Koskinas 1925; Sarkisov et al. 1949) and intersubject variability.

FG1 is located on the medial half of the posterior fusiform gyrus and extends rostrally on the lateral bank of the collateral sulcus. FG2 is located lateral to FG1 on the lateral half of the fusiform gyrus reaching into the lateral occipitotemporal sulcus.

An interpretation of the cytoarchitectonical areas described here should be based on a comparison with previously proposed cytoarchitectonical maps and results of recent functional imaging research. It should be noted, however, that a simple comparison of center of gravity coordinates is problematic because of tremendous divergences between the coordinates in the different functional imaging reports [e.g. Bartels and Zeki (2004) report the center of the FFA at L: [−44, −46, −24]/R: [44, −46, −26], while Spiridon et al. (2006) report L: [−50.1, −69.2, −7.5]/R: [31.3, −55.8, −5.9], both labeled “Talairach space”). The divergences may be caused by the usage of different reference spaces, since the terms “MNI space” or “Talairach space” are not sufficiently clear definitions in many studies. Thus, the stereotaxic coordinates of the same cortical site can vary considerably between the different studies even if the same definition, e.g. “MNI space” was used, depending on the precise position of the CA-CP-line (CA = commissura anterior, CP = commissura posterior) and other aspects as shown in detail by Lancaster et al. (2007). Due to this remarkable lack of comparability between studies using seemingly identical reference space, the purely coordinate-based correlation between cytoarchitectonical and functional imaging data coming from different studies is problematic, particularly if positions of cortical areas have to be compared. In this case, minimal differences in the position of the CA-CP-line lead to rather large deviations in location of the rather remote cortical areas. Therefore, a comparison of stereotaxic coordinates has to be critically weighted against a combination of topographic descriptions, neighborhood relations and reported illustrations.

### Comparison with previous architectonic maps

Classical anatomical maps of the human brain show a tripartition of the visual cortex (Brodmann 1909; von Economo and Koskinas 1925; Sarkisov et al. 1949). Brodmann (1909) mentioned that his temporal area BA37, which adjoins BA19 in its ventral parts, is a transitional area between temporal and occipital regions. Von Economo and Koskinas (1925) found a roughly comparable area to BA37 adjoining their area O_A_, which can be seen as an equivalent to BA19. They describe the cytoarchitecture of this adjoining area as highly heterogeneous but were not able to delimit clearly defined subregions of this area. Because they saw primarily cytoarchitectonical characteristics of the parietal lobe, they assigned this area to the parietal region and labeled it “area parietalis temporooccipitalis” or P_H_. Whereas the classical architectonical definitions of the primary and secondary visual cortex (BA17 and BA18) are fully agreed by most observers (Amunts et al. 2000), it became evident by functional neuroimaging and non-human primate data that BA19/O_A_ should be subdivided into multiple functionally and putatively also architectonically distinct regions (Zeki 1969; van Essen 1979; Braak 1980; Tootell et al. 1996). It seems conceivable that FG1 and FG2 are situated within the border region between BA19 and BA37 or O_A_ and P_H_, respectively.

Detailed maps of the occipital and adjacent temporo-parietal lobe were published by Braak (1977) in his pigmentoarchitectonical study. His report included drawings of identified areas on the cortical surface as well as coronal sections, which makes a comparison less difficult. FG1 and FG2 topographically fit to Braak’s (1977) “area peristriata densopyramidalis”. Its description as a “well-developed bitaeniate cortex with conspicuous pIIIc” (“bitaeniate” = “double-striped”) is likewise in accordance with our observations on the cytoarchitecture of area FG2. Braak (1977), however, identified a single area in this region. Our results indicate clear cytoarchitectonical differences between two areas and hence a sub-parcellation of “area peristriata densopyramidalis” into FG1 and FG2.

### Comparison with functional imaging data

*Retinotopy* is a common organizational principle of early visual areas in the primate visual cortex (e.g. Sereno et al. 1995; DeYoe et al. 1996; Engel et al. 1997). Utilizing this principle, retinotopic mapping has emerged to the gold standard in functional imaging of these early visual areas and yielded robust results for the borders of areas V1, V2 and V3v (Tootell et al. 1998; Wade et al. 2002; Wohlschläger et al. 2005).

However, there has been an intense debate during the last decade concerning the anterior border of human area V4. One view proposed an area V4v, representing the (contralateral) upper-quarterfield, followed anteriorly by a color-selective area, containing a complete hemifield representation termed V8 (Hadjikhani et al. 1998; Tootell and Hadjikhani 2001). This arrangement was challenged by others, who postulated V3v to be the most anterior quarterfield representation, which is then followed by a hemifield representation selective for color perception. They labeled this area the V4-complex, containing V4 and an anterior non-retinotopic subarea V4α. The authors did not see any evidence for a quarterfield representation like in V4v (Bartels and Zeki 1998, 2000; Zeki 2001). A third scheme contained an upper field representation, analogous to V4v, followed anteriorly by its matching lower field termed human V4 (hV4). The authors argued for yet another color-selective hemifield representation, VO-1 anterior to it (Wade et al. 2002; Wandell et al. 2005; Brewer et al. 2005).

It should be mentioned that the hV4/VO-1 model does not contradict the V4-complex model, but can be harmonized with it, while the V8 model cannot (Wade et al. 2002). Furthermore, the hV4/VO-1 model was later confirmed by other laboratories (Kastner et al. 2001; Larsson et al. 2006; Arcaro et al. 2009; Kolster et al. 2010). Our study shows two distinct cytoarchitectonical areas FG1 and FG2 antero-lateral to the cytoarchitectonical area hOc4v (Rottschy et al. 2007), which overlaps with retinotopically defined hV4 (Wilms et al. 2010). The descriptions of VO-1 as an area in the collateral sulcus and on the medial fusiform gyrus (Brewer et al. 2005; Liu and Wandell 2005) would topographically fit to FG1. However, the apparently closer proximity of VO-1 to the collateral sulcus and its location directly anterior to hV4 as well as the reported coordinates (Kastner et al. 2001; Brewer et al. 2005; Liu and Wandell 2005; Arcaro et al. 2009) suggest that VO-1 is more medial than FG1 and probably correlates with the medially adjoining cytoarchitectonical area col.s.* (Fig. 8c).

Recently, another retinotopic region has been discovered lateral to hV4, i.e. the phPIT cluster (Kolster et al. 2010), which refers to macaque areas PITd and PITv on the posterior inferotemporal gyrus (Felleman and van Essen 1991). The phPIT cluster is located on the inferior temporal gyrus at the posterior end of the lateral occipitotemporal sulcus and consists of two hemifield representations, phPITd and phPITv, which share their foveal representation and vertical meridians. The lateral of our identified cytoarchitectonical areas, FG2, is located on the lateral bank of the posterior fusiform gyrus and within the posterior lateral occipitotemporal sulcus. FG2 rarely reaches the inferior temporal gyrus. Thus, the topography and the center of gravity coordinates indicate that phPIT is located posterior and dorsal to FG2, and most probably overlaps with the laterally adjoining cortex (Fig. 8d).

Taken together, the comparison with current retinotopic literature indicates that FG1 and FG2 do not correspond to any hitherto identified retinotopic area. Instead, both cytoarchitectonical areas seem to fill in the “non-retinotopic gap” that is spanned between VO-1 medially and phPITv laterally (see Kolster et al. 2010, Fig. 16A). A minor peripheral overlap between these retinotopic and our cytoarchitectonical areas cannot be completely ruled out. However, such a mismatch between cytoarchitectonically and retinotopically defined cortical units would be in contrast to the correlation of both parcellation approaches in other cortical areas, e.g. in V1 and V2 (Wohlschläger et al. 2005).

Besides its retinotopic organization, the human ventral visual cortex contains a number of apparently *category-specific functional modules* for visual object processing. Some regions that respond more strongly to visual objects than to scrambled images were identified around the posterior fusiform gyrus and the inferior occipital gyrus. They are called the “lateral occipital complex” (LOC, Malach et al. 1995, 2002; Kanwisher et al. 1996; Grill-Spector et al. 2001). Bilateral LOC-activations receive input from both hemifields (Grill-Spector et al. 1998) and are correlated with recognition performance of objects (Grill-Spector et al. 2000). Conventionally, the LOC comprises two entities, the dorsal “LO” and the ventral and anterior “pFs” on the posterior and mid-fusiform gyrus, which differ in their response to size and position changes of presented objects (Grill-Spector et al. 1999). A later scheme assigned pFs to an object-selective cluster “VOT” of the ventral occipitotemporal cortex directly adjoining early retinotopic visual areas (Malach et al. 2002). In consideration of the pertinent reports of the LOC, a direct correspondence of our areas FG1 and FG2 to a LOC-cluster seems to be unlikely, as they are located between both classical clusters, ventral to LO and posterior to pFs. Indeed, the center of gravity of FG2 is in close proximity to the coordinates of the “branching point” between both LOC-clusters described by Malach et al. (1995). Thus, an overlap of the margins of LOC with FG2 is possible. Based on the VOT-scheme predicting object-responsive areas directly adjacent to early visual areas (Malach et al. 2002), it can be hypothesized that FG1 and FG2 are both situated within the higher order object-related cortex.

Additional to the LOC and partially overlapping with it, some areas selective for specific objects have been identified within the ventral occipitotemporal cortex including the prototypical example of the “fusiform face area” (FFA). First hints on the existence of the FFA were derived from studies on subjects with prosopagnosia. These subjects showed a bilateral or right-hemispheric lesion in the ventral occipitotemporal cortex (Damasio et al. 1982; De Renzi 1986; Landis et al. 1986; Sergent and Signoret 1992). Later PET studies revealed a distinct activation on the fusiform gyrus during face perception tasks in healthy volunteers (Sergent et al. 1992; Haxby et al. 1994). The face specificity and location of the FFA could be demonstrated at higher spatial resolution in numerous fMRI studies (Clark et al. 1996; Puce et al. 1996; Kanwisher et al. 1997; Tong et al. 1998; Halgren et al. 1999; Hasson et al. 2001; Grill-Spector et al. 2004; Kanwisher and Yovel 2006). In addition, a response to images of headless bodies was reported on the fusiform gyrus in the vicinity of the FFA (Cox et al. 2004; Peelen and Downing 2005). High resolution fMRI identified this activation as adjacent yet distinct from the FFA in the fusiform body area (FBA, Schwarzlose et al. 2005). Most functional investigations locate the FFA on the lateral bank of the posterior or mid-fusiform gyrus, about 1–2 cm anterior to the here identified cytoarchitectonical area FG2 (e.g. Gauthier et al. 2000; Hasson et al. 2001; Levy et al. 2001; Avidan et al. 2003; Rossion et al. 2003; Grill-Spector et al. 2004; Bartels and Zeki 2004; Peelen and Downing 2005). However, some reports show peak activations much closer to FG2 (Puce et al. 1996; Kanwisher et al. 1997; Halgren et al. 1999; Ishai et al. 1999). This discrepancy might be explained by a recent investigation of the fusiform gyrus demonstrating a subdivision of the FFA into a posterior and an anterior part (Weiner and Grill-Spector 2010, 2011a), which was also suggested by others (Pinsk et al. 2009; Mei et al. 2010). The posterior face patch named pFus-faces accurately matches the location of our lateral cytoarchitectonical area FG2 situated on the lateral bank of the posterior fusiform gyrus antero-lateral to hV4 and, hence, might be its functional correlate.

Another functional, category-specific area, which is located within the lateral occipitotemporal sulcus extending onto the lateral fusiform gyrus is the visual word-form area (VWFA), which responds specifically to words and letter strings. First hints came from patients suffering from lesions in the ventral occipitotemporal cortex and respective neuropsychological deficits, i.e. pure alexia (Damasio and Damasio 1983; Binder and Mohr 1992). This functionally defined area could later be localized by PET (Petersen et al. 1990; Petersen and Fiez 1993), MEG (Tarkiainen et al. 1999) and fMRI (Wagner et al. 1998; Cohen et al. 2000; Hasson et al. 2002; Dehaene et al. 2002), although the functional specificity of VWFA was also controversially discussed (Price and Devlin 2003). Similar to our area FG2, the VWFA was first described to be located on the lateral fusiform gyrus (Cohen et al. 2000; Dehaene et al. 2002), but more recent findings show that the large portion of VWFA lies within the fundus of the lateral occipitotemporal sulcus and about 1 cm anterior to FG2 (Cohen and Dehaene 2004; Baker et al. 2007; Ben-Shachar et al. 2007; Wandell et al. 2012). An overlap of FG2 with language-related visual areas is possible, since FG2 largely extends into the lateral occipitotemporal sulcus and distances of FG2 to the center of gravity of VWFA are quite small (e.g. Cohen et al. 2002; Vigneau et al. 2005; Dehaene et al. 2010; Mei et al. 2010). Moreover, the processing of words seems to continuously extend from early visual areas to the ventral occipitotemporal locations in a hierarchical manner (Dehaene et al. 2005; Szwed et al. 2011), where FG2 could possibly be involved at an intermediate stage.

Our results indicate that FG1 and FG2 are symmetrically found in all ten brains, showing the same cytoarchitectonical features on both sides and no significant left–right differences in volume. By contrast, the majority of reports show a right lateralization of the FFA that apparently depends on handedness (Willems et al. 2010), while the VWFA is most often left lateralized (e.g. Cohen and Dehaene 2004). This does not exclude a possible overlap of the functional and our cytoarchitectonical areas, since different functional manifestations can be implemented on the same cytoarchitectonical basis. Furthermore, recent investigations imply that lateralization for faces (Weiner and Grill-Spector 2010) and words (Ben-Shachar et al. 2007) is possibly much less pronounced as hitherto assumed.

Thus, our cytoarchitectonical areas FG1 and FG2 probably lie within the object-related higher order visual cortex on the posterior fusiform gyrus. Furthermore, FG2 likely comprises a posterior fusiform face-selective patch. However, distinct functional correlates in this region are rare. This issue might also be affected by the fMRI artifacts evoked by the transverse sinus, a venous vessel, which commonly proceeds directly inferior to the cortex we investigated here (Winawer et al. 2010). Appropriately, Winawer et al. (2010) denote this region as ‘No man’s land’. A more specific-functional characterization of FG1 and FG2 and the relation to object, face and visual word processing remains a topic for future work. It can certainly be encouraged by further improved functional imaging techniques and comprehensive approaches including cytoarchitectonical, retinotopic and category-related investigations. For now, we provide probability maps that can be used to relate functional measurements to our cytoarchitectonical delineations and, hence, can help to understand the functional role of this region rostrally adjoining the early ventral visual cortex.

## Conclusions

The present study detected two novel, cytoarchitectonically distinct areas on the human posterior fusiform gyrus, using a quantitative microscopical approach. The delineation of the extrastriate areas is used to provide probabilistic maps in standardized 3D-stereotaxic space. The maps allow a comparison of functional imaging data on object, face and word-form recognition with its putative cytoarchitectonical correlates. Comparisons between our map and previous functional imaging studies suggest that FG1 and FG2 are situated in a not yet retinotopically mapped gap between VO-1 and phPITv whereas FG2 is the cytoarchitectonical correlate of the posterior face patch pFus-faces.

## References

[CR1] Amunts K, Zilles K (2001). Advances in cytoarchitectonic mapping of the human cerebral cortex. Neuroimaging Clin N Am.

[CR2] Amunts K, Malikovic A, Mohlberg H, Schormann T, Zilles K (2000). Brodmann’s areas 17 and 18 brought into stereotaxic space-where and how variable?. Neuroimage.

[CR3] Amunts K, Weiss PH, Mohlberg H, Pieperhoff P, Eickhoff S, Gurd JM, Marshall JC, Shah NJ, Fink GR, Zilles K (2004). Analysis of neural mechanisms underlying verbal fluency in cytoarchitectonically defined stereotaxic space—the roles of Brodmann areas 44 and 45. Neuroimage.

[CR4] Amunts K, Kedo O, Kindler M, Pieperhoff P, Mohlberg H, Shah NJ, Habel U, Schneider F, Zilles K (2005). Cytoarchitectonic mapping of the human amygdala, hippocampal region and entorhinal cortex: intersubject variability and probability maps. Anat Embryol (Berl).

[CR5] Annett M (1973). Handedness in families. Ann Hum Genet.

[CR6] Arcaro MJ, McMains SA, Singer BD, Kastner S (2009). Retinotopic organization of human ventral visual cortex. J Neurosci.

[CR7] Avidan G, Levy I, Hendler T, Zohary E, Malach R (2003) Spatial vs. object specific attention in high-order visual areas. Neuroimage 19(2 Pt 1):308–318 10.1016/s1053-8119(03)00092-2 12814581

[CR8] Baker CI, Liu J, Wald LL, Kwong KK, Benner T, Kanwisher N (2007). Visual word processing and experiential origins of functional selectivity in human extrastriate cortex. Proc Natl Acad Sci USA.

[CR9] Bartels PH (1979). Numerical evaluation of cytologic data: II. Comparison of profiles. Anal Quant Cytol.

[CR10] Bartels A, Zeki S (1998). The theory of multistage integration in the visual brain. Proc Biol Sci.

[CR11] Bartels A, Zeki S (2000). The architecture of the colour centre in the human visual brain: new results and a review. Eur J Neurosci.

[CR12] Bartels A, Zeki S (2004). Functional brain mapping during free viewing of natural scenes. Hum Brain Mapp.

[CR13] Ben-Shachar M, Dougherty RF, Deutsch GK, Wandell BA (2007). Differential sensitivity to words and shapes in ventral occipito-temporal cortex. Cereb Cortex.

[CR14] Binder JR, Mohr JP (1992). The topography of callosal reading pathways. A case-control analysis. Brain.

[CR15] Braak H (1977). The pigment architecture of the human occipital lobe. Anat Embryol (Berl).

[CR16] Braak H (1980). Architectonics of the human telencephalic cortex.

[CR17] Brewer AA, Liu J, Wade AR, Wandell BA (2005). Visual field maps and stimulus selectivity in human ventral occipital cortex. Nat Neurosci.

[CR18] Brodmann K (1909). Vergleichende Lokalisationslehre der Großhirnrinde.

[CR19] Caspers S, Eickhoff SB, Geyer S, Scheperjans F, Mohlberg H, Zilles K, Amunts K (2008). The human inferior parietal lobule in stereotaxic space. Brain Struct Funct.

[CR20] Clark VP, Keil K, Maisog JM, Courtney S, Ungerleider LG, Haxby JV (1996). Functional magnetic resonance imaging of human visual cortex during face matching: a comparison with positron emission tomography. Neuroimage.

[CR21] Clarke S, Miklossy J (1990). Occipital cortex in man: organization of callosal connections, related myelo- and cytoarchitecture, and putative boundaries of functional visual areas. J Comp Neurol.

[CR22] Cohen L, Dehaene S (2004). Specialization within the ventral stream: the case for the visual word form area. Neuroimage.

[CR23] Cohen L, Dehaene S, Naccache L, Lehericy S, Dehaene-Lambertz G, Henaff MA, Michel F (2000). The visual word form area: spatial and temporal characterization of an initial stage of reading in normal subjects and posterior split-brain patients. Brain.

[CR24] Cohen L, Lehericy S, Chochon F, Lemer C, Rivaud S, Dehaene S (2002). Language-specific tuning of visual cortex? Functional properties of the visual word form area. Brain.

[CR25] Cox D, Meyers E, Sinha P (2004). Contextually evoked object-specific responses in human visual cortex. Science.

[CR26] Damasio AR, Damasio H (1983). The anatomic basis of pure alexia. Neurology.

[CR27] Damasio AR, Damasio H, Van Hoesen GW (1982). Prosopagnosia: anatomic basis and behavioral mechanisms. Neurology.

[CR28] De Renzi E (1986). Prosopagnosia in two patients with CT scan evidence of damage confined to the right hemisphere. Neuropsychologia.

[CR29] Dehaene S, Le Clec HG, Poline JB, Le Bihan D, Cohen L (2002). The visual word form area: a prelexical representation of visual words in the fusiform gyrus. NeuroReport.

[CR30] Dehaene S, Cohen L, Sigman M, Vinckier F (2005). The neural code for written words: a proposal. Trends Cogn Sci.

[CR31] Dehaene S, Pegado F, Braga LW, Ventura P, Nunes Filho G, Jobert A, Dehaene-Lambertz G, Kolinsky R, Morais J, Cohen L (2010). How learning to read changes the cortical networks for vision and language. Science.

[CR32] DeYoe EA, Carman GJ, Bandettini P, Glickman S, Wieser J, Cox R, Miller D, Neitz J (1996). Mapping striate and extrastriate visual areas in human cerebral cortex. Proc Natl Acad Sci USA.

[CR33] Downing PE, Jiang Y, Shuman M, Kanwisher N (2001). A cortical area selective for visual processing of the human body. Science.

[CR34] Eickhoff SB, Stephan KE, Mohlberg H, Grefkes C, Fink GR, Amunts K, Zilles K (2005). A new SPM toolbox for combining probabilistic cytoarchitectonic maps and functional imaging data. Neuroimage.

[CR35] Eickhoff SB, Amunts K, Mohlberg H, Zilles K (2006). The human parietal operculum. II. Stereotaxic maps and correlation with functional imaging results. Cereb Cortex.

[CR36] Eickhoff SB, Paus T, Caspers S, Grosbras MH, Evans AC, Zilles K, Amunts K (2007). Assignment of functional activations to probabilistic cytoarchitectonic areas revisited. Neuroimage.

[CR37] Eickhoff SB, Rottschy C, Kujovic M, Palomero-Gallagher N, Zilles K (2008). Organizational principles of human visual cortex revealed by receptor mapping. Cereb Cortex.

[CR38] Engel SA, Glover GH, Wandell BA (1997). Retinotopic organization in human visual cortex and the spatial precision of functional MRI. Cereb Cortex.

[CR39] Epstein RA (2008). Parahippocampal and retrosplenial contributions to human spatial navigation. Trends Cogn Sci.

[CR40] Epstein R, Harris A, Stanley D, Kanwisher N (1999). The parahippocampal place area: recognition, navigation, or encoding?. Neuron.

[CR41] Evans AC, Marrett S, Neelin P, Collins L, Worsley K, Dai W, Milot S, Meyer E, Bub D (1992). Anatomical mapping of functional activation in stereotactic coordinate space. Neuroimage.

[CR42] Felleman DJ, van Essen DC (1991). Distributed hierarchical processing in the primate cerebral cortex. Cereb Cortex.

[CR43] Gauthier I, Tarr MJ, Moylan J, Skudlarski P, Gore JC, Anderson AW (2000). The fusiform “face area” is part of a network that processes faces at the individual level. J Cogn Neurosci.

[CR44] Grill-Spector K, Kushnir T, Hendler T, Edelman S, Itzchak Y, Malach R (1998). A sequence of object-processing stages revealed by fMRI in the human occipital lobe. Hum Brain Mapp.

[CR45] Grill-Spector K, Kushnir T, Edelman S, Avidan G, Itzchak Y, Malach R (1999). Differential processing of objects under various viewing conditions in the human lateral occipital complex. Neuron.

[CR46] Grill-Spector K, Kushnir T, Hendler T, Malach R (2000). The dynamics of object-selective activation correlate with recognition performance in humans. Nat Neurosci.

[CR47] Grill-Spector K, Kourtzi Z, Kanwisher N (2001). The lateral occipital complex and its role in object recognition. Vision Res.

[CR48] Grill-Spector K, Knouf N, Kanwisher N (2004). The fusiform face area subserves face perception, not generic within-category identification. Nat Neurosci.

[CR49] Hadjikhani N, Liu AK, Dale AM, Cavanagh P, Tootell RB (1998). Retinotopy and color sensitivity in human visual cortical area V8. Nat Neurosci.

[CR50] Halgren E, Dale AM, Sereno MI, Tootell RB, Marinkovic K, Rosen BR (1999). Location of human face-selective cortex with respect to retinotopic areas. Hum Brain Mapp.

[CR51] Hasson U, Hendler T, Ben Bashat D, Malach R (2001). Vase or face? A neural correlate of shape-selective grouping processes in the human brain. J Cogn Neurosci.

[CR52] Hasson U, Levy I, Behrmann M, Hendler T, Malach R (2002). Eccentricity bias as an organizing principle for human high-order object areas. Neuron.

[CR53] Haxby JV, Horwitz B, Ungerleider LG, Maisog JM, Pietrini P, Grady CL (1994). The functional organization of human extrastriate cortex: a PET-rCBF study of selective attention to faces and locations. J Neurosci.

[CR54] Hömke L (2006). A multigrid method for anisotrophic PDE’s in elastic image registration. Numer Linear Algebra Appl.

[CR55] Ishai A, Ungerleider LG, Martin A, Schouten JL, Haxby JV (1999). Distributed representation of objects in the human ventral visual pathway. Proc Natl Acad Sci USA.

[CR56] Kanwisher N, Yovel G (2006). The fusiform face area: a cortical region specialized for the perception of faces. Philos Trans R Soc Lond B Biol Sci.

[CR57] Kanwisher N, Chun MM, McDermott J, Ledden PJ (1996). Functional imaging of human visual recognition. Brain Res Cogn Brain Res.

[CR58] Kanwisher N, McDermott J, Chun MM (1997). The fusiform face area: a module in human extrastriate cortex specialized for face perception. J Neurosci.

[CR59] Kastner S, De Weerd P, Pinsk MA, Elizondo MI, Desimone R, Ungerleider LG (2001). Modulation of sensory suppression: implications for receptive field sizes in the human visual cortex. J Neurophysiol.

[CR60] Kolster H, Peeters R, Orban GA (2010). The retinotopic organization of the human middle temporal area MT/V5 and its cortical neighbors. J Neurosci.

[CR61] Kurth F, Eickhoff SB, Schleicher A, Hoemke L, Zilles K, Amunts K (2010). Cytoarchitecture and probabilistic maps of the human posterior insular cortex. Cereb Cortex.

[CR62] Lancaster JL, Tordesillas-Gutierrez D, Martinez M, Salinas F, Evans A, Zilles K, Mazziotta JC, Fox PT (2007). Bias between MNI and Talairach coordinates analyzed using the ICBM-152 brain template. Hum Brain Mapp.

[CR63] Landis T, Cummings JL, Christen L, Bogen JE, Imhof HG (1986). Are unilateral right posterior cerebral lesions sufficient to cause prosopagnosia? Clinical and radiological findings in six additional patients. Cortex.

[CR64] Larsson J, Landy MS, Heeger DJ (2006). Orientation-selective adaptation to first- and second-order patterns in human visual cortex. J Neurophysiol.

[CR65] Levy I, Hasson U, Avidan G, Hendler T, Malach R (2001). Center-periphery organization of human object areas. Nat Neurosci.

[CR66] Liu J, Wandell BA (2005). Specializations for chromatic and temporal signals in human visual cortex. J Neurosci.

[CR67] Mahalanobis PC, Majumdar DN, Rao CR (1949). Anthropometric survey of the United Provinces, 1941: a statistical study. Sankya.

[CR68] Malach R, Reppas JB, Benson RR, Kwong KK, Jiang H, Kennedy WA, Ledden PJ, Brady TJ, Rosen BR, Tootell RB (1995). Object-related activity revealed by functional magnetic resonance imaging in human occipital cortex. Proc Natl Acad Sci USA.

[CR69] Malach R, Levy I, Hasson U (2002). The topography of high-order human object areas. Trends Cogn Sci.

[CR70] Malikovic A, Amunts K, Schleicher A, Mohlberg H, Eickhoff SB, Wilms M, Palomero-Gallagher N, Armstrong E, Zilles K (2007). Cytoarchitectonic analysis of the human extrastriate cortex in the region of V5/MT+: a probabilistic, stereotaxic map of area hOc5. Cereb Cortex.

[CR71] Mei L, Xue G, Chen C, Xue F, Zhang M, Dong Q (2010). The “visual word form area” is involved in successful memory encoding of both words and faces. Neuroimage.

[CR72] Merker B (1983). Silver staining of cell bodies by means of physical development. J Neurosci Methods.

[CR73] Mishkin M, Ungerleider LG (1982). Contribution of striate inputs to the visuospatial functions of parieto-preoccipital cortex in monkeys. Behav Brain Res.

[CR74] Orban GA, Van Essen D, Vanduffel W (2004). Comparative mapping of higher visual areas in monkeys and humans. Trends Cogn Sci.

[CR75] Peelen MV, Downing PE (2005). Selectivity for the human body in the fusiform gyrus. J Neurophysiol.

[CR76] Petersen SE, Fiez JA (1993). The processing of single words studied with positron emission tomography. Annu Rev Neurosci.

[CR77] Petersen SE, Fox PT, Snyder AZ, Raichle ME (1990). Activation of extrastriate and frontal cortical areas by visual words and word-like stimuli. Science.

[CR78] Pinsk MA, Arcaro M, Weiner KS, Kalkus JF, Inati SJ, Gross CG, Kastner S (2009). Neural representations of faces and body parts in macaque and human cortex: a comparative FMRI study. J Neurophysiol.

[CR79] Price CJ, Devlin JT (2003). The myth of the visual word form area. Neuroimage.

[CR80] Puce A, Allison T, Asgari M, Gore JC, McCarthy G (1996). Differential sensitivity of human visual cortex to faces, letterstrings, and textures: a functional magnetic resonance imaging study. J Neurosci.

[CR81] Rossion B, Schiltz C, Crommelinck M (2003). The functionally defined right occipital and fusiform “face areas” discriminate novel from visually familiar faces. Neuroimage.

[CR82] Rottschy C, Eickhoff SB, Schleicher A, Mohlberg H, Kujovic M, Zilles K, Amunts K (2007). Ventral visual cortex in humans: cytoarchitectonic mapping of two extrastriate areas. Hum Brain Mapp.

[CR83] Sarkisov SA, Filimonoff IN, Preobrashenskaya NS (1949). Cytoarchitecture of the human cortex cerebri.

[CR84] Scheperjans F, Eickhoff SB, Hömke L, Mohlberg H, Hermann K, Amunts K, Zilles K (2008). Probabilistic maps, morphometry, and variability of cytoarchitectonic areas in the human superior parietal cortex. Cereb Cortex.

[CR85] Schleicher A, Amunts K, Geyer S, Morosan P, Zilles K (1999). Observer-independent method for microstructural parcellation of cerebral cortex: a quantitative approach to cytoarchitectonics. Neuroimage.

[CR86] Schleicher A, Amunts K, Geyer S, Kowalski T, Schormann T, Palomero-Gallagher N, Zilles K (2000). A stereological approach to human cortical architecture: identification and delineation of cortical areas. J Chem Neuroanat.

[CR87] Schleicher A, Palomero-Gallagher N, Morosan P, Eickhoff SB, Kowalski T, de Vos K, Amunts K, Zilles K (2005). Quantitative architectural analysis: a new approach to cortical mapping. Anat Embryol (Berl).

[CR88] Schleicher A, Morosan P, Amunts K, Zilles K (2009). Quantitative architectural analysis: a new approach to cortical mapping. J Autism Dev Disord.

[CR89] Schwarzlose RF, Baker CI, Kanwisher N (2005). Separate face and body selectivity on the fusiform gyrus. J Neurosci.

[CR90] Sereno MI, Dale AM, Reppas JB, Kwong KK, Belliveau JW, Brady TJ, Rosen BR, Tootell RB (1995). Borders of multiple visual areas in humans revealed by functional magnetic resonance imaging. Science.

[CR91] Sergent J, Signoret JL (1992). Varieties of functional deficits in prosopagnosia. Cereb Cortex.

[CR92] Sergent J, Ohta S, MacDonald B (1992). Functional neuroanatomy of face and object processing. A positron emission tomography study. Brain.

[CR93] Spiridon M, Fischl B, Kanwisher N (2006). Location and spatial profile of category-specific regions in human extrastriate cortex. Hum Brain Mapp.

[CR94] Szwed M, Dehaene S, Kleinschmidt A, Eger E, Valabregue R, Amadon A, Cohen L (2011). Specialization for written words over objects in the visual cortex. Neuroimage.

[CR95] Tarkiainen A, Helenius P, Hansen PC, Cornelissen PL, Salmelin R (1999). Dynamics of letter string perception in the human occipitotemporal cortex. Brain.

[CR96] Tong F, Nakayama K, Vaughan JT, Kanwisher N (1998). Binocular rivalry and visual awareness in human extrastriate cortex. Neuron.

[CR97] Tootell RB, Hadjikhani N (2001). Where is ‘dorsal V4’ in human visual cortex? Retinotopic, topographic and functional evidence. Cereb Cortex.

[CR98] Tootell RB, Dale AM, Sereno MI, Malach R (1996). New images from human visual cortex. Trends Neurosci.

[CR99] Tootell RB, Mendola JD, Hadjikhani NK, Liu AK, Dale AM (1998). The representation of the ipsilateral visual field in human cerebral cortex. Proc Natl Acad Sci USA.

[CR100] Tootell RB, Tsao D, Vanduffel W (2003). Neuroimaging weighs in: humans meet macaques in “primate” visual cortex. J Neurosci.

[CR101] Ungerleider LG, Haxby JV (1994). ‘What’ and ‘where’ in the human brain. Curr Opin Neurobiol.

[CR102] van Essen DC (1979). Visual areas of the mammalian cerebral cortex. Annu Rev Neurosci.

[CR103] Vigneau M, Jobard G, Mazoyer B, Tzourio-Mazoyer N (2005). Word and non-word reading: what role for the visual word form area?. Neuroimage.

[CR104] von Economo C, Koskinas GN (1925). Die Cytoarchitektonik der Hirnrinde des Erwachsenen Menschen.

[CR105] Wade AR, Brewer AA, Rieger JW, Wandell BA (2002). Functional measurements of human ventral occipital cortex: retinotopy and colour. Philos Trans R Soc Lond B Biol Sci.

[CR106] Wagner AD, Schacter DL, Rotte M, Koutstaal W, Maril A, Dale AM, Rosen BR, Buckner RL (1998). Building memories: remembering and forgetting of verbal experiences as predicted by brain activity. Science.

[CR107] Wandell BA, Brewer AA, Dougherty RF (2005). Visual field map clusters in human cortex. Philos Trans R Soc Lond B Biol Sci.

[CR108] Wandell BA, Rauschecker AM, Yeatman JD (2012). Learning to see words. Annu Rev Psychol.

[CR109] Weiner KS, Grill-Spector K (2010). Sparsely-distributed organization of face and limb activations in human ventral temporal cortex. Neuroimage.

[CR110] Weiner KS, Grill-Spector K (2011). Neural representations of faces and limbs neighbor in human high-level visual cortex: evidence for a new organization principle. Psychol Res.

[CR111] Weiner KS, Grill-Spector K (2011). Not one extrastriate body area: using anatomical landmarks, hMT+, and visual field maps to parcellate limb-selective activations in human lateral occipitotemporal cortex. Neuroimage.

[CR112] Willems RM, Peelen MV, Hagoort P (2010). Cerebral lateralization of face-selective and body-selective visual areas depends on handedness. Cereb Cortex.

[CR113] Wilms M, Eickhoff SB, Hömke L, Rottschy C, Kujovic M, Amunts K, Fink GR (2010). Comparison of functional and cytoarchitectonic maps of human visual areas V1, V2, V3d, V3v, and V4(v). Neuroimage.

[CR114] Winawer J, Horiguchi H, Sayres RA, Amano K, Wandell BA (2010). Mapping hV4 and ventral occipital cortex: the venous eclipse. J Vis.

[CR115] Wohlschläger AM, Specht K, Lie C, Mohlberg H, Wohlschläger A, Bente K, Pietrzyk U, Stöcker T, Zilles K, Amunts K, Fink GR (2005). Linking retinotopic fMRI mapping and anatomical probability maps of human occipital areas V1 and V2. Neuroimage.

[CR116] Zeki SM (1969). Representation of central visual fields in prestriate cortex of monkey. Brain Res.

[CR117] Zeki S (2001). Localization and globalization in conscious vision. Annu Rev Neurosci.

[CR118] Zilles K, Amunts K (2010). Centenary of Brodmann’s map—conception and fate. Nat Rev Neurosci.

[CR119] Zilles K, Clarke S (1997) Architecture, connectivity and transmitter receptors of human extrastriate visual cortex: comparison with non-human primates. In: Rockland KS, Kaas JH, Peters A (eds) Cerebral cortex, vol 12. Plenum Press, New York, London, pp 673–742

[CR120] Zilles K, Schleicher A, Palomero-Gallagher N, Amunts K, Mazziotta JC, Toga A (2002). Quantitative analysis of cyto- and receptor architecture of the human brain. Brain mapping: the methods.

